# Risk factors and outcomes of acute kidney injury in ventilated newborns

**DOI:** 10.1080/0886022X.2019.1665546

**Published:** 2019-11-07

**Authors:** Yuanyuan Fan, Jinkun Ye, Lijuan Qian, Ruibin Zhao, Ning Zhang, Liwen Xue, Lixing Qiao, Li Jiang

**Affiliations:** a Department of Pediatrics, Zhongda Affiliated Hospital of Southeast University, Nanjing, China;; b Department of Pediatrics, Nanjing Maternal and Child Health Hospital, Nanjing, China;; c Department of Pediatrics, Tian Kang Hospital, Tianchang, China;; d Department of Pediatrics, The First Hospital of Changzhou, Changzhou, China

**Keywords:** Mechanical ventilation, acute kidney injury, newborn, risk factor, prognosis

## Abstract

**Purpose:** This study aimed to investigate the occurrence and risk factors of acute kidney injury (AKI) in ventilated newborns.

**Methods:** In total, 139 newborns receiving mechanical ventilation (MV) were reviewed in this retrospective study. The demographic and clinical data were collected. Then, the independent risk factors for AKI were evaluated using univariate and multivariate logistic regression analyses.

**Results:** The incidence rate of AKI was 15.11% (21/139) in ventilated newborns. Univariate analysis showed significant differences in gestational age, birth weight, Apagar scores, the highest oxygen concentration, serum creatinine levels at admission and 48 h after MV, history of asphyxia, urine output at 48 h after MV, invasive MV, noninvasive MV, and outcomes between AKI and non-AKI groups (all *p* < .05). The lower gestational age (odd ratio (OR): 1.194, 95% confidence interval (CI): 1.013–1.407, *p* = .035), the increased use of invasive mechanical ventilation (IMV) (OR: 4.790, 95% CI: 1.115–20.575, *p* = .035), and lower birth weight (OR: 0.377, 95% CI: 0.178–0.801, *p* = .011) were independent risk factors for the occurrence of AKI. Additionally, higher stage of AKI was significantly associated with poor prognosis of AKI (*p* = .018).

**Conclusion:** In this retrospective study, it was found that lower gestational age, birth weight, and increased use of IMV were independent risk factors for AKI in ventilated newborns. The poor prognosis might be indicated by the higher AKI stage.

## Introduction

Acute kidney injury (AKI) is a common severe disease in the intensive care unit (ICU), defined by an acutely increased level of serum creatinine (SCr) or/and a reduced urine output (UO) according to the acute kidney injury network (AKIN) criteria [1,2]. It has been reported that the incidence of AKI was 18–52% in critically ill pediatric patients, and patients with AKI have a higher risk of poor prognosis [3]. Thus, it is imperative to understand the risk factors of AKI and search for effective intervention and therapy for pediatric patients with AKI.

Mechanical ventilation (MV) is an auxiliary ventilation way to improve the acute respiratory distress syndrome and often applied in the neonatal intensive care unit (NICU), especially for preterm infants [4]. However, MV with high or low tidal volume after acid aspiration can differentially affect the changes in systemic organs inflammation, suggesting the different effects of MV strategy on organ dysfunction [5]. Previous study had shown that MV contributes to the development of AKI, which may be associated with arterial blood gases or lung injury caused by ventilation induced release of inflammatory factors [6]. The high incidence of AKI has been demonstrated in critically ill newborns, and results in poor short- and long-term outcomes [7]. Currently, studies on the AKI in ventilated newborn infants are rarely reported. In neonatal population, peculiar renal pathophysiology, lower glomerular filtration rate, and the influence from maternal SCr as well as other factors make it difficult to diagnose AKI; in addition, the occurrence of AKI in newborns was caused by manifold contributing factors [8]. Hence, we designed this study to investigate the occurrence and risk factors of AKI in ventilated newborns.

The present study reviewed the baseline characteristics and outcomes of ventilated newborns and evaluated the incidence rate of AKI defined by AKIN criteria in ventilated newborns. Furthermore, the risk factors of AKI in ventilated newborns were investigated to research for effective interventions in reducing the poor prognosis and mortality rates.

## Material and methods

### Study population

This retrospective study was approved by the Ethics Committee of Zhongda Hospital Affiliated to Southeast University (Ethical approval number: 2019ZDSYLL079-901). According to the inclusion and exclusion criteria, a total of 139 newborns (94 boys and 45 girls) who admitted after birth to the NICU of Department of Pediatrics, Zhongda Hospital Affiliated to Southeast University between January 2014 and December 2014 were included in this study. The inclusion criteria were: newborns (1) received MV, including invasive (MV due to intubation is called invasive MV) and noninvasive, for at least 1 day after initial admission; (2) received the determinations of SCr for at least two times within the 48 h; and (3) had a detailed record of urine at least every 4 h. Newborns, who were dead within the 48 h, had severe urinary tract malformation or kidney damage before MV, or suffered with cardiac diseases, as well patients with cardiac disease or a syndromic diagnosis were excluded in this study. Demographic and clinical data, including gender, postnatal age, birth weight, gestational age, Apagar score, the history of asphyxia, the length of stay, UO at 48 h after MV, SCr levels at admission and after MV, and pH, *P*O_2_, *P*CO_2_, K+, and lactate levels at admission and after the occurrence of AKI, as well as information on MV, such as invasive mechanical ventilation (IMV), noninvasive mechanical ventilation (NIMV), the highest oxygen concentration, the time of NIMV, and whether received pulmonary surfactant, vancomycin were collected. UO was measured according to the diaper weight. In addition, the management of fluid balance was performed in accordance with the guidelines for clinical application of nutritional support for newborns in China. Furthermore, the prognosis of patients were recorded as follows: death, improved (without obvious symptom, but required re-examination), and cured (the clinical symptoms completely disappeared and had no any abnormal physical signs).

### Study definitions

AKI was defined following the AKIN criteria and newborns were divided into AKI and non-AKI groups. Categorical definition of neonatal AKI was that: AKI-1 stage, increased SCr ≥26.5 μmol/L (0.3 mg/dL) or a percentage increase of SCr ≥150–200% within the 48 h; AKI-2 stage, a percentage increase of SCr ≥ 200–300% within the 48 h; and AKI-3 stage, increased SCr ≥221.0 μmol/L (2.5 mg/dL) or a percentage increase of SCr ≥300% within the 48 h or need for dialysis.

### Statistical analysis

All statistical analyses were carried out using the standard statistical package of SPSS 17.0 (IBM, Armonk, New York, USA). The normality test of the continuous variables was performed using Shapiro–Wilk method. Normally distributed data were presented as mean ± standard deviation (SD) and the non-normal distribution data were expressed as median and interquartile range. All categorical variables were presented as frequency. The significance of difference between the AKI and non-AKI groups was analyzed using univariate logistic regression analysis. Furthermore, the independent risk factors for AKI were evaluated using multivariate logistic regression analysis. A value with *p* < .05 was considered as statistically significant difference.

## Results

### Patient characteristics

In total, 139 ventilated newborn and the determinations of SCr for at least two times within the 48 h were reviewed. The incidence rate of AKI was 15.11% (21/139). Among the 21 newborns with AKI, there were 14 cases with AKI-1, 4 cases with AKI-2, and 3 cases with AKI-3. Of the 139 newborns, 55 cases (39.57%) received IMV, 109 cases (78.42%) received NIMV, and 25 cases (17.99%) received both IMV and NIMV.

### The risk factors of AKI in ventilated newborn

Baseline characteristics and outcomes were compared between AKI and non-AKI groups (Table 1). Univariate analysis showed significant differences in gestational age, birth weight, Apagar scores, the highest oxygen concentration, SCr level at admission and 48 h after MV, UO at 48 h after MV, history of asphyxia, IMV, NIMV, and outcomes between AKI and non-AKI groups (all *p* < .05, Table 1), suggesting that the occurrence of AKI was associated with the lower gestational age, lower birth weight, Apagar scores, the highest oxygen concentration, without the history of asphyxia, lower SCr level before MV, higher SCr level at 48 h after MV, the decreased UO at 48 h after MV, the increased use of IMV, and the reduced use of NIMV. Furthermore, the multivariate logistic regression analysis (Table 2) showed that the lower gestational age (odd ratio (OR): 1.194, 95% confidence interval (CI): 1.013–1.407, *p* = .035), the increased use of IMV (OR: 4.790, 95% CI: 1.115–20.575, *p* = .035), and lower birth weight (OR: 0.377, 95% CI: 0.178–0.801, *p* = .011) were independent risk factors for the occurrence of AKI.

**Table 1.** Univariate logistic regression analysis of baseline characteristics and outcomes between acute kidney injury (AKI) and non-AKI groups.

Factors	AKI (*n* = 21)	Non-AKI (*n* = 118)	*p* value
Lactate levels at admission (mmol/L)	1.80 (1.50–2.60)	1.90 (1.40–3.60)	.52
Lactate levels after AKI (mmol/L)	2.40 (1.45–4.30)	2.00 (1.18–2.90)	.16
pH at admission	7.35 ± 0.08	7.36 ± 0.10	.78
pH after AKI	7.41 ± 0.07	7.40 ± 0.09	.75
*P*O_2_ at admission (mmHg)	74.90 (50.45–87.25)	73.35 (51.38–89.88)	.52
*P*O_2_ after AKI (mmHg)	72.50 (57.00–79.15)	70.90 (55.88–81.23)	1.00
*P*CO_2_ at admission (mmHg)	38.80 (35.00–47.30)	39.70 (35.10–48.30)	.94
*P*CO_2_ after AKI (mmHg)	38.33 ± 9.32	38.90 ± 9.73	.80
K + at admission	4.00 (3.55–4.55)	4.30 (3.90–5.20)	.18
K + after AKI	4.00 (3.35–4.45)	4.05 (3.40–4.60)	.82
The length of stay (day)	8.0 (4.5–12.0)	11.5 (8.0–17.0)	.14
Postnatal age (h)	1.50 (1.00–12.25)	2.00 (1.50–7.00)	.38
Gestational age (week)	33.00 (30.50–35.00)	35.00 (32.00–38.00)	.03
Birth weight (g)	2016.19 ± 698.35	2555.38 ± 866.53	.01
Apagar scores (1 min)	7.0 (5.0–9.0)	9.0 (7.0–9.0)	.008
Apagar scores from 0–3	3 (14.3%)	5 (4.2%)	.013
Apagar scores from 4 to 7	9 (42.9%)	26 (22.0%)	
Apagar scores from 8 to 10	9 (42.9%)	87 (73.7%)	
Apagar scores (5 min)	8.0 (8.0–9.0)	9.0 (9.0– 9.0)	.007
Apagar scores from 4 to 7	5 (23.8%)	12 (10.2%)	.105
Apagar scores from 8 to 10	16 (76.2%)	106 (89.8%)	
The time of MV from birth (h)	2.0 (1.0–3.0)	2.0 (2.0–3.0)	.489
The highest oxygen concentration	0.35 (0.28–0.70)	0.30 (0.25–0.40)	.02
Urine volume before MV (mL/h/kg)	2.38 ± 1.30	2.79 ± 0.82	.171
Urine volume 48 h after MV (mL/h/kg)	2.29 ± 1.31	2.84 ± 0.97	.03
SCr level at admission (μmol/L)	46.00 (40.50–54.50)	57.00 (47.75–67.00)	.02
SCr level 48 h after MV (μmol/L)	134.00 (113.50–190.00)	73.00 (57.75–87.25)	<.01
The time of NIMV (day)	3.50 (2.00–8.75)	3.00 (2.00–4.00)	.31
Gender			
Male	13 (61.90%)	81 (68.64%)	.544
Female	8 (38.10%)	37 (31.36%)	
The history of asphyxia			
Yes	13 (61.91%)	38 (32.20%)	.01
No	8 (38.10%)	77 (65.25%)	
Use of pulmonary surfactant			
Yes	3 (14.29%)	16 (13.56%)	.93
No	18 (85.71%)	102 (86.44%)	
Use of Vancomycin			
Yes	1 (4.76%)	10 (8.47%)	.887
No	20 (95.24%)	108 (91.53%)	
IMV			
Yes	16 (76.19%)	39 (33.05%)	<.01
No	5 (23.81%)	79 (66.95%)	
NIMV			
Yes	10 (47.62%)	99 (83.90%)	<.01
No	11 (52.38%)	19 (16.10%)	
Outcome			
Abandon treatment	5 (23.81%)	9 (7.63%)	.048
Improved cases	8 (38.10%)	35 (29.66%)	
Death	3 (14.29%)	10 (8.47%)	
Cured cases	5 (23.81%)	64 (54.24%)	

MV: mechanical ventilation; NIMV: noninvasive mechanical ventilation; IMV: invasive mechanical ventilation.

**Table 2.** The multivariate logistic regression analysis of risk factors for the occurrence of acute kidney injury (AKI).

Factors	*p* value	OR	95%CI
IMV	.035	4.790	1.115–20.575
NIMV	.221	0.359	0.070–1.854
The history of asphyxia	.964	0.957	0.144–6.378
The highest oxygen concentration	.995	1.010	0.057–17.758
Birth weight	.011	0.377	0.178–0.801
Gestational age	.035	1.194	1.013–1.407
Apagar scores (1 min)	.872	0.894	0.228–3.504

MV: mechanical ventilation; NIMV: noninvasive mechanical ventilation; IMV: invasive mechanical ventilation; OR: odd ratio; CI: confidence interval.

### Effect of AKI stage on prognosis

Among the 21 newborns with AKI, 3 cases (14.29%, including 2 cases with AKI-2 and 1 case with AKI-3) were dead, 13 cases (61.90%, including 9 cases with AKI-1, 2 cases with AKI-2 and 2 cases with AKI-3) had improved therapeutic effect, and 5 cases (23.81%) with AKI-1 were cured (Table 3). The higher stage of AKI was related to the poor prognosis (*p* = .02, Table 3).

**Table 3.** The prognosis of newborns with different stages of acute kidney injury (AKI).

AKI stage	Prognosis			*p* value
	Death (*n* = 3)	Improved (*n* = 13)	Cured (*n* = 5)	
1	0	9	5	.02
2	2	2	0	
3	1	2	0	

## Discussion

AKI is a common clinical illness in newborns and could be induced by various diseases, such as heart failure, asphyxia, and sepsis [9]. Notably, MV was also reported to be an important risk factor for AKI [10]. The present study found that AKI occurred in 15.11% of ventilated newborns, and lower gestational age, lower birth weight, Apagar scores, the highest oxygen concentration, without the history of asphyxia, lower SCr level before MV, higher SCr level at 48 h after MV, the decreased UO at 48 h after MV, the increased use of IMV, and the reduced use of NIMV were related to the occurrence of AKI. Furthermore, the lower gestational age, lower birth weight, and the increased use of IMV were considered as independent risk factors for the occurrence of AKI. Moreover, higher stage of AKI indicated a poor prognosis.

Currently, AKI is often diagnosed by the ‘Risk, Injury, Failure, Loss, End-Stage Kidney Disease (RIFLE)’ criteria or AKIN criteria. Several pediatric studies have validated the applications of both the pediatric RIFLE and AKIN criteria in children [11–14]. Compared with RIFLE criteria, AKIN criteria increased the detection rate of AKI, while no significant difference was found in predicting mortality of critically ill patients [12]. Currently, few studies have applied the AKIN criteria in the newborn population. This study evaluated the incidence rate of AKI in ventilated newborns according to the AKIN criteria. Previous study had shown almost 20% of AKI using UO and SCr criteria in NICU [15], while another study demonstrated only 6.3% of AKI diagnosed by only SCr criteria in newborn patients [16]. In the present study, AKI was defined by only the increase of SCr and a higher incidence rate (15.11%) was found in ventilated newborns which had to be ventilated because of hypoxia and that was what caused AKI as it takes a day or two for creatinine to rise. Furthermore, this study revealed that patients who diagnosed with the higher AKI stage according to the AKIN criteria had the worse prognosis in ventilated newborns. It was reported that AKI severity was closely correlated with the length of hospital stay as well as the early and late mortality [12], nevertheless, that was not confirmed in our study.

At present, AKI diagnosis was mainly based on an acute increase of SCr levels or a decrease of UO, that were considered as late effects of injury, and in neonatal population, peculiar renal pathophysiology, lower glomerular filtration rate, and the influence from maternal SCr as well as other factors make it difficult to diagnose AKI [8]. Neonatal non-oliguric AKI is generally considered probably due to higher total body water, especially in preterm infants (up to 80%), and immature tubular cells might be a main cause for the water content difference between newborns and other populations [17]. Therefore, the identification of new biomarkers for the early AKI diagnosis was encouraged. Urine interleukin-6 was considered as an early biomarker of AKI because that there was an increase of urine interleukin-6 at six hours in AKI patients, that probably caused by proximal tubule injury [18]. In addition, urine albumin [19], neutrophil gelatinase-associated lipocalin [20], interleukin-18 [21], and other were all reported to be urine biomarker of AKI.

It is well-known that the occurrence of AKI in newborns is caused by manifold contributing factors, including sepsis, asphyxia as well as congestive heart failure, lung failure, dehydration, and nephrotoxic drugs [22,23]. The present study focused on the factors that influenced the occurrence of AKI in ventilated newborns. The ventilated newborn had a higher risk of bronchopulmonary dysplasia, poor neurodevelopment, and renal injury [6,24,25]. Van Marter et al. suggested that judicious use of inspired oxygen and peak inspiratory pressure might decrease the risk of chronic lung disease [25]. Oxygen had acute and chronic toxic effects on organs such as lungs and brain. It affected blood circulation, endocrine function, enzyme system, and molecular structure [26]. High fraction of inspired oxygen influenced surfactant synthesis, and the produced excess oxygen free radicals might attack organs and overwhelm the defense systems [27]. Consistently, we found that the highest oxygen concentration was implicated in the occurrence of AKI in ventilated newborns. Several studies compared the effects of IMV and NIMV on AKI and revealed that patients with IMV had higher risk of AKI than those with NIMV [28–30]. A recent meta-analysis had also demonstrated that IMV might be a risk factor for AKI in the critically ill patients [31]. Shaffer et al. [32] further showed that NIMV might prevent the pathophysiologic effects compared with IMV. Consistently, this study revealed that increased use of IMV but not NIMV was closely associated with the occurrence of AKI in the NICU. In addition, this study found that the lower birth weight was associated with the occurrence of AKI in ventilated newborns. A case–control study had demonstrated that AKI might increase the mortality of very low birth weight newborns [33]. Another study also suggested low birth weight newborns had the elevated risk of AKI [34]. The high incidences of oliguria and asphyxia were also demonstrated in neonatal AKI [9], which was consistent with our study. Moreover, this study also revealed that the gestational age and IMV were risk factors for the occurrence of AKI in ventilated newborns. Askenazi et al. found that baseline values of candidate urine AKI biomarkers vary by gestational age in premature infants [27].

Lower gestational age, lower birth weight, and increased use of IMV were found to be independent risk factors for AKI in ventilated newborns in this study, and some potential risk factors associated with AKI were found. However, the limitation of this study was the retrospective nature and small sample size. Thus, the results of this study should be further confirmed by a prospective study with larger sample size.

## Conclusion

This study revealed that AKI occurred in 15.11% of ventilated newborns, and the poor prognosis could be indicated by the higher AKI stage. Furthermore, the occurrence of AKI was associated with several factors, including the lower gestational age, lower birth weight, Apagar scores, without the history of asphyxia, lower SCr level before MV, higher SCr level at 48 h after MV, the decreased UO at 48 h after MV, the increased use of IMV, and the reduced use of NIMV. Specifically, the lower gestational age, lower birth weight, and the increased use of IMV were independent risk factors for AKI in ventilated newborns.
